# Emerging Role of *Eruca sativa* Mill. in Male Reproductive Health

**DOI:** 10.3390/nu16020253

**Published:** 2024-01-14

**Authors:** Dhekra Grami, Slimen Selmi, Kais Rtibi, Hichem Sebai, Luca De Toni

**Affiliations:** 1 Laboratory of Functional Physiology and Valorization of Bioresources-Higher Institute of Biotechnology of Beja, University of Jendouba, Beja B.P. 382-9000, Tunisia; slimen.selmi@gmail.com (S.S.); rtibikais@yahoo.fr (K.R.); sebaihichem@yahoo.fr (H.S.); 2 Department of Medicine and Unit of Andrology and Reproductive Medicine, University of Padova, Via Giustiniani 2, 35128 Padova, Italy

**Keywords:** *Eruca sativa*, semen parameters, oxidative stress, spermatogenesis, testosterone

## Abstract

A growing interest has been drawn to the use of traditional medicinal plants for the treatment of human diseases and, in particular, infertility and reproductive toxicity associated with environmental factors. The Mediterranean basin area is a recognized source of plant species with therapeutic interest. In this frame, *Eruca sativa* (ES) is an annual edible plant and a member of the Brassicaceae family. A relatively large number of studies, focusing on the biological effects of the extract from the leaves of ES on in vitro and in vivo models of disease, have been published in recent years. The present narrative review aims to analyze the phytochemical constituents, traditional uses, possible pharmacological activities, and recognized effects of ES on male reproductive outcomes. Available investigations have revealed the presence of a number of compounds with antioxidant properties, such as polyphenols, glucosinolates, flavonoids, and carotenoids in extracts from ES. Based on the chemical and pharmacological characteristics of the aforementioned compounds, we show that ES has possible preventive properties and therapeutic uses, especially in the functional derangements of the male reproductive system.

## 1. Introduction

Aside from supplying vital nutrients essential for sustaining life, food also serves as the primary carrier of various bioactive compounds able to promote health and prevent diseases [1]. Substantial correlations have currently been established between the adoption of a correct dietary regimen and the reduced risk of health issues such as neurodegenerative disorders, cardiovascular diseases, cancer, diabetes, inflammation, and reproductive disorders [2]. In this frame, traditional medicine encompasses a multitude of herbal remedies suggested to play a role in the prevention and treatment of the aforementioned clinical conditions.

Infertility, or sub-fertility, is a multifactorial clinical condition defined as the reduced or delayed ability of a couple to have childhood despite having unprotected sexual intercourses. Infertility can be attributed to either couple, female, or male factors, the latter being largely related to defects in spermatogenesis, namely the process of differentiation from spermatogonial germ cells to mature spermatozoa [3].

*Brassica* L., is a genus of plants in the Brassicaceae family, which includes herbaceous plants with large leaves, some of which have great economic importance for human activities. Plants belonging to this family are highly valued for their nutritional properties, including the relative abundance of compounds with antioxidant properties, including polyphenols. These compounds are known not only for the flavor they impart to the food but also for their recognized health benefits, mainly related to their aforementioned antioxidant properties [4]. *Eruca sativa* Mill. (ES), in particular, is a very popular species of the *Brassicaceae* family widely cultivated in the area of the Mediterranean basin. In this narrative review, existing evidence concerning the customary applications, phytochemical profile, the pharmacological activity, and toxicological studies regarding ES have been summarized. To achieve this aim, available full-text papers in the English language from PubMed (PubMed (https://pubmed.ncbi.nlm.nih.gov/)) and Google Scholar (Google Scholar, https://scholar.google.com/) databases, involving the use of Eruca Sativa in animal or human models and evaluating testicular function and/or histology, were taken into consideration. Moreover, the plant’s possible use as a remedy to prevent or treat heath issues, in particular, male reproductive disorders, is discussed.

## 2. *Eruca sativa*: Taxonomic Framework and Phytochemical Characterization

The *Eruca* (Miller) and *Diplotaxis* (DC) genera is commonly known as arugula rocket leaves or Jarjeer in Arabic [5,6]. Other traditional names include rucola, rucoli, rugula, roquette, and pokain in Greek, according to the country. The plant has been recognized since ancient times and was documented in the Greek herbal of Material Medical [7]. It is an annual plant, reaching heights of up to 1 m, characterized by dark green leaves typically measuring less than 20 cm in length. The basal leaves form a rosette and are lobed to pinnatifid, while the upper leaves of the plant are pinnatifid, featuring long-oblong terminal lobes that can be either coarsely toothed or lobed [8]. Rocket is a collective term for plants that produce rosettes of vividly green, divided leaves with a distinctive flavor. The genus *Eruca* L. consists of a single species, *Eruca vesicaria* (L.) Cav., with three infraspecific taxa: subsp. *Sativa* (Miller) Thell., subsp. *Vesicaria*, and subsp. *Pinnatifida* (Desf.) Emberger et Maire [9]. *E. vesicaria* subsp. *sativa* (Miller) Thell. is commonly referred to by its simplified synonym *E. sativa* Mill. It represents the only taxon with a fairly broad distribution around the Mediterranean region, including the Maghreb nations in North Africa, including Tunisia, Algeria, and Morocco [9]. These species are currently cultivated worldwide in countries ranging from the USA to UK, Italy, Spain, Morocco, to even India and Australia [10]. Overall, optimal growth in a temperate climate and extensive cultivation for food purposes make ES a widely available plant at the global level.

The nutritional supply of ES has been studied at different levels of qualitative and quantitative detail. In terms of macronutrients, ES leaves are a source of carbohydrate fibers, magnesium, calcium, and sodium, whereas the seeds are a source of fats, phosphorus, calcium, sodium, potassium, and magnesium [11]. ES leaves are also rich in vitamins, including carotenes, tocopherols, vitamin C, and folic acid. In general, whole ES is a source of vitamins A, C, and K, as well as thiamin, riboflavin, niacin, vitamin B-6 (pyridoxine), and pantothenic acid [12].

The essential oil fraction extracted from the leaves, known to confer the typical organoleptic properties with little or no biological effects of note, has been characterized. It is notably rich in nitrogen and sulfur-containing compounds, including isothiocyanate and 5-methylthiopentyl isothiocyanate [13]. On the other hand, is has been demonstrated that ES seed oil comprises 93.8% fatty acids, with 6.7% of these being saturated. The more detailed composition includes erucic acid (46.6–54.8%), oleic acid (17.9–19.9%), palmitic acid (7.3–10.9%), linoleic acid (4.2–9.7%), and linolenic acid (1.9–3.0%) [14]. Environmental stressors are known to possibly influence the concentration of the individual components. However, they do not result in an increase in the yield of these compounds per unit area [15].

**Figure 1:**
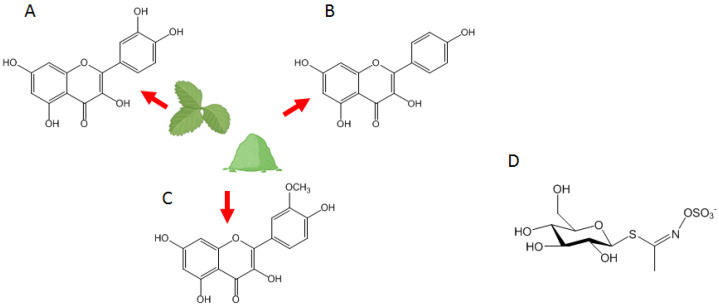
Prominent flavonoids in ES include: (**A**) Quercetin; (**B**) Kaempferol; (**C**) Isorhamnetin; and (**D**) the structure of glucosinolates.

Higher importance for the biological activity of ES is provided by the leaf composition in terms of glucosinolates (GSL) and flavonoids [16]. In particular, 4-methylsulfinylbutyl GLS (glucoraphanin), 4-(β-D-glucopyranosyldisulfanyl) butyl GLS (diglucothiobeinin), 4-hydroxylglucobrassicin, GLS (4-hydroxylglucobrassicin) 4-mercaptobutyl GLS (glucosativin), 4-methylthiobutyl GLS (glucoerucin), 4-mercaptobutyl GLS dimer, and 1-methoxy-3-indolylmethyl GLS (neoglucobrassicin) have been identified [17]. In seeds and roots, the major GSLs were found to be DS-glucoraphanin and DS-glucoerucin. The nitrile 5-methylthiopentanenitrile has been observed as one of the constituents in the volatile fraction of ES. However, nitriles, thiocyanates, and isothiocyanates are considered degradation products of glucosinolates [18] (Figure 1). Overall, the estimated total content of GSL in ES ranges from 14.0 to 28.2 µM/g of dry weight [19].

Finally, various studies have aimed to quantify polyphenols in ES leafs, primarily by High-Performance Liquid Chromatography approaches. Major compounds of the flavonoid family are kaempferol, isorhamnetin, rhamnocitrin, quercetin, and their glycosides [20] (Figure 1). Within the crude organic extract of both the aerial parts and roots of ES, campesterol, β-sitosterol, and brassicasterol emerge as the most prevalent sterols. Triterpenes, predominantly β-amyrin, were also identified, but at lower concentrations [21]. Phenolic acids, such as Chlorogenic acid, Caffeic acid, and Ferulic acid, and flavonols, such as Isorhamnetin, Quercetin, and Kaempferol, have also been described [21].

## 3. Traditional Uses, and Pharmacological and Toxicological Profiles of *Eruca sativa*

The use of ES was mainly borne for food consumption in aerial parts or as a spice in either leaves, seeds, or flowers. The peculiar aroma of rocket is a consequence of the presence of glucosinolates: glucoerucin, glucoraphanin, gluociberin, and glucocochlearin. It is typically enjoyed fresh in salads and as a cooked green, and is now very popular as a pizza topping [22]. However, ES is utilized beyond its culinary applications. In the Indian subcontinent, the cultivation of specific ecotypes of ES is dedicated to seed production and subsequently used in the extraction of oil [23]. Since Roman times, it has also gained popularity for its reputed aphrodisiac effects [22] and is regarded as a medicinal plant with a multitude of reported properties [24,25,26]. In traditional medicine, the leaves of ES have been extensively used as a remedy for various digestive problems, including use as a carminative, to alleviate abdominal discomfort, and to improve digestion by local herbal medicine practitioners. In this context, the leaves are employed in traditional medicine for their astringent, diuretic, digestive, emollient, tonic, depurative, laxative, rubefacient, stimulant, and antimicrobial properties [27,28]. In Arabian countries, it is a traditional practice to use the seeds and tender leaves of ES to enhance sexual desire, deeming them aphrodisiacs. Studies have indicated that the widely used ethanolic extract of ES seeds exhibits notable properties as renal protective and diuretic [28,29]. Additionally, it has been documented that ES exhibits anti-hyperlipidemic, anti-hyperglycemic, and hepato-protective properties [30,31]. Most of the available studies on ES suggest that the major pharmacological activity relies on the antioxidant effects [32] resulting, in turn, in some ameliorative effects on diseases whose pathogenesis associates with a redox imbalance, including inflammation [20], cancer [21], platelet aggregation, thrombosis [33], gastric lesions [6], allergies and hypersensitivity [34], and nociception [19,35].

From a toxicological point of view, a tentative evaluation of the *ES* median lethal dose (LD_50_) was provided by Abdulla Salih et al. in 2022, aiming to address the possible protective effect of ES leaves extract on doxorubicin-induced cardiotoxicity in rabbits [36]. The estimated LD_50_ value of ES leaves’ ethanolic extract, evaluated through an up-and-down approach upon 24 h of exposure to animals [37], was 3129.5 mg/kg, suggesting the broad safety of the whole plant for food purposes in usual domestic portions.

## 4. Regulation of Spermatogenesis and Risk Factors of Male Infertility

**Figure 2:**
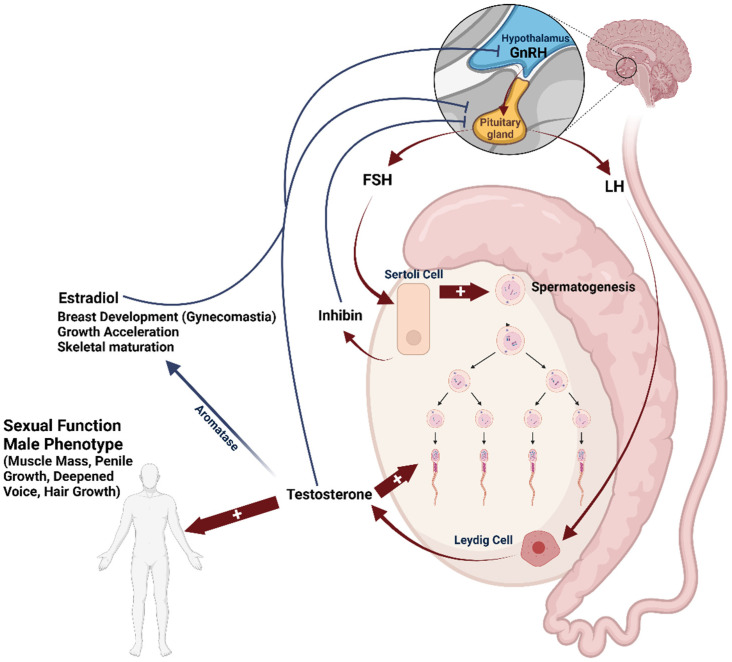
Hormonal regulation of testis function through the hypothalamus-pituitary-gonadal (HPG) axis, involving the hypothalamic gonadotropin-releasing hormone (GnRH)-dependent production of pituitary gonadotropins, follicle stimulating hormone (FSH), and luteinizing hormone (LH). In turn, testosterone is produced by Leydig cells under LH control. FSH, together with testosterone, promotes the correct process of spermatogenesis. Testosterone also supports the development of male sexual traits. The aromatase enzyme is involved in testosterone conversion into estradiol, the main estrogen, which owns specific physiological (and pathogenic) roles in males. The HPG axis undergoes negative feedback control by sex steroids and by inhibin released by Sertoli cells.

The endocrine control of spermatogenesis has been a widely explored topic in several scientific disciplines, including developmental biology, veterinary, clinical medicine, and cell biology. Gonadal function is mainly regulated by the hormonal activity within the hypothalamic-pituitary-gonadal (HPG) axis (summarized in Figure 2).

The correct development and maintenance of testis function depend on the release of pituitary gonadotropins: Follicle-Stimulating Hormone (FSH), and Luteinizing Hormone (LH), in response to the hypothalamic gonadotropin-releasing hormone (GnRH), which is produced by the anterior pituitary gland in a pulsatile manner. FSH and LH exert their specific effects through their respective receptors: Follicle-Stimulating Hormone Receptor (FSHR) and Luteinizing Hormone Receptor (LHR). Both receptors are G-protein coupled receptors and are expressed by Sertoli cells within the seminiferous tubule and by the interstitial Leydig cells population, respectively [38,39]. In turn, LH stimulates the production of testosterone (T), which is essential for sperm production and maturation, as well as for the development of secondary sexual characteristics and anabolic functions [40,41]. On the other hand, FSH promotes the proliferation and sustenance of Sertoli cell function, which is essential for sperm maturation [42,43]. In particular, FSH is recognized to target the initial stages of spermatogenesis until the entry of germ cells into the meiosis phase. Subsequently, working in tandem with T, FSH triggers signaling pathways within Sertoli cells, facilitating the maturation of germ cells [44,45,46]. This process also encompasses the provision of antiapoptotic factors for cell survival and the regulation of adhesion complexes between germ cells and Sertoli cells [47]. The pituitary secretion of FSH and LH is controlled by a negative feedback mechanism involving gonadal sex steroids and inhibin produced by Sertoli cells. These substances collectively inhibit the secretion of GnRH, and help maintain the homeostasis of the HPG axis [48,49]. Following the onset of puberty, spermatogenesis typically persists without any interruption throughout an individual’s lifespan, although there may be seasonal fluctuations in certain animal species. However, a gradual decline of serum T is documented from the fourth decade of life onwards due to a progressive reduction in testicular steroidogenic function and/or reduced pituitary gonadotropin secretion [50].

Male fertility primarily hinges on factors such as sperm count, quality, motility, and morphology. Thus, any impairment in these aspects is associated with male infertility. A recent investigation demonstrated that spermatozoa are particularly susceptible to oxidative stress due to the high presence of polyunsaturated fatty acids in cell membranes [51]. The correct redox balance within the urogenital tract is sustained by a range of antioxidants that both spermatozoa and seminal plasma possess, in order to neutralize reactive oxygen species (ROS). These include enzymes like superoxide dismutase (SOD), catalase (CAT), the glutathione peroxidase/reductase system, as well as various compounds like alpha-tocopherol, ascorbic acid, glutathione, pyruvate, taurine, hypo-taurine, and albumin itself [52,53]. The primary origins of endogenous ROS identified in semen are leukocytes and defective spermatozoa [54,55,56]. In this frame, several authors have suggested that low levels of ROS are required to maintain significant function in regular physiological processes such as sperm capacitation, hyperactivation, and acrosome reaction [57,58]. However, in pathological conditions, the unbalanced over-production of ROS severely affects sperm function and the overall fertilization process. This is due to its detrimental effects on the spermatogenesis process, as well as on various aspects of sperm function and structure, including mobility, viability, acrosome reaction, sperm-to-oocyte binding, and may even lead to reduced fertilization and implantation rates [59,60,61,62,63,64].

It is crucial to note that elevated ROS levels in the urogenital tract can stem from various lifestyle factors, such as excessive smoking, alcohol consumption, and environmental influences like radiation and bacterial exposure [65]. Moreover, metabolic diseases like diabetes have been associated with reproductive issues in both partners. In fact, individuals with diabetes often experience sexual problems, including decreased libido, impotence, and infertility [66,67]. On the other hand, altered sperm parameters are frequently observed in diabetic patients [68]. The effects of diabetes-related hyperglycemia on the HPG axis are wide and currently under investigation. Hassan et al. [68] observed that the reduction in testosterone levels in diabetic rats was the likely the result of compromised Leydig cell function. The absence of a rise in serum LH levels associated with low T levels in the untreated diabetic group was attributed to a disruption in the feedback mechanism. Accordingly, diabetes dampened the LH response to gonadectomy in both male and female rats [68]. In addition, glycemic overload has been demonstrated to exert direct effects on cell mitochondria. According to this model, in somatic cells, the excessive commitment of the respiratory chain due to hyperglycemia was associated with the cellular accumulation of reduced equivalents such as NADH and uncontrolled over-production of ROS [69]. Interestingly, this pathophysiological mechanism has also been found in spermatozoa [70]. As proof of the pathogenetic role of oxidative stress on testis function, lifestyle modifications such as weight loss, physical activity, and stopping of alcohol and smoking, have proven to be highly effective in improving the markers of infertility associated with metabolic disorders [71]. In this regard, the intake of a diet rich in fruit and vegetables is often adopted as a countermeasure to improve the intake of natural antioxidants, such as tocopherols, carotenes, and flavonoids, capable of scavenging excess ROS [72]. Although still a subject of controversy, the use of food supplements based on natural antioxidants is also commonly adopted as a therapy against male infertility. Accordingly, several studies provide evidence about the effectiveness of this approach in improving both pregnancy and fertilization rates in patients with male factors of infertility [73,74,75].

## 5. Effects of *Eruca sativa* on Male Fertility: Data from Animal Studies

In traditional oriental medicine, various herbs have been employed since ancient times to enhance male sexual function [76]. Being a source of polyphenols with antioxidant properties, ES shows a rationale for its use in the treatment of pathologies associated with oxidative stress, including male infertility. Indeed, a substantial body of evidence from animal studies indicates a favorable effect of the administration of ES extracts or oil in different animal models of male reproductive disorders.

Pioneer data were provided by Salem and Moustafa in 2001, using albino rats as animal model, to test the possible urogenital toxicity of ES seed oil orally administered at different dosages, three times per week for six weeks [77]. The authors reported that at the lowest tested dose of 0.25 mL/kg, the treatment with ES seed oil was associated with a significant increase in the proliferation of the haploid germ cell population compared to untreated control animals. Conversely, higher doses were associated with hypospermatogenesis and markers of reduced DNA synthesis, evaluated by sperm count and at the histological level, respectively. Similar results were obtained more recently by Hussein et al. in the same animal model, testing the effect of the aqueous extract of ES leaves [78]. Compared to control animals, the sub-chronic administration of 30 and 40 mg/kg body weight of the ES extract over a period of 5 weeks was associated with a significant increase in T levels, together with a reduction in the percentage of non-viable sperms and cells with morphological abnormalities. Still regarding the aqueous extract of ES, in a preliminary report in 2014, Ansari and Ganaie showed that the altered sperm parameters found in the diabetic mouse model, induced by streptozotocin exposure, were significantly improved by the administration of ES extract at doses of 250 and 500 mg/kg for 8 consecutive weeks [79]. Interestingly, the weight of testes, epididymis, seminal vesicles, and prostate were also significantly increased at the end of the treatment compared to untreated diabetic animals. A tentative report using an animal model of reproductive derangement was that from Hassan and Meligi, in which male rats exposed to abamectin, a known neurotoxic pesticide, were evaluated [80]. In this study, abamectin exposure was associated with a significant imbalance of the HPG axis, with severe T reduction and strong alteration of testis histology. Oral administration of the ES fresh whole plant water suspension, 5 g/kg body weight every 48 h for 28 days, was able to partly restore T levels and testis tissue architecture.

Other studies explored the possible effect of ES on known environmental factors associated with male reproductive derangements. In 2016, Abd El-Aziz et al. assessed the possible effect of ES seed oil (0.25 mL/kg/day) in 8-week-old rats exposed to nicotine at a dosage of 2.5 mg/kg/day, corresponding to “heavy smoker” exposure. After a four-week treatment, compared to unexposed controls, nicotine exposure resulted in significantly reduced body weight gain and, particularly, reduced absolute and relative testis weight [81]. Interestingly, this finding was associated with a significant reduction in serum T and severe alterations in testis histology, such as the reduction of the mean seminiferous tubule diameter and thinning of seminiferous epithelium height. On the other hand, treatment with ES seed oil, while having no significant effect on anthropometry and testicular outcome when administered alone, was associated with a significant improvement in all the aforementioned parameters in nicotine-exposed animals [81]. Despite these interesting results, however, the authors did not provide experimental proof of the possible mechanism subtending the evident protective role of ES. In addition to nicotine, cigarette smoke is an acknowledged source of exposure to several other toxicants, including heavy metals. In this regard, a certain tropism towards testicular tissue has been found for Cadmium, probably due to ionic mimicry with other metal ions, such as zinc and cadmium, of major importance in the physiology of the male gonad [82]. In 2016, Al-Okaily and Al-Shammari investigated the possible direct toxicity of oral Cadmium on the Leydig cell compartment of the testis in an adult rat model exposed to oral Cadmium chloride for up to 56 days, at a concentration of 30 mg/L administered through tap water [83]. The authors found that from the 28th day of exposure to Cadmium onwards, animals showed a significant depression of the whole HPG axis, resulting in a significant reduction in serum T, LH, and FSH compared to unexposed controls. This hormonal evidence was associated with a significant reduction in the number of Leydig cells upon histological evaluation. Interestingly, the concomitant treatment with ES ethanolic extract at a daily dose of 250 mg/Kg was associated with an early and significant recovery of serum T and LH levels after 28 days of treatment, followed by a later increase in FSH levels at the 56th day of treatment. Even in this case, the authors did not provide mechanistic proof of ES’s effect, limiting themselves to hypothesizing a possible role in reducing oxidative stress [83]. However, it should be noted that, given the different toxico-dynamics of Cadmium, a possible chelating effect of ES polyphenols on heavy metals, resulting in reduced absorption of these latter, cannot be excluded [84].

In line with the aforementioned in vivo data, recent findings from Grami et al. showed the possible involvement of reduced oxidative stress as the underlying mechanism supporting the improvement in semen parameters associated with the administration of ES leaves’ aqueous extract at low doses [85]. In particular, authors used a peculiar animal model of reproductive derangement, namely male rats exposed to bisphenol A (BPA) [85]. BPA is a known technological adjuvant used for plastics manufacturing, and its role as an endocrine disruptor has been largely investigated [86]. In animal models, gestational exposure to BPA has been associated with sex developmental disorders in newborns, while in adults, BPA exposure has been associated with hormonal derangements, impaired spermatogenesis. and altered sperm parameters [87]. Accordingly, Grami et al. reported that, compared to control animals, 4 weeks of exposure to 100 mg/kg/day of BPA was associated with reduced serum T, severely reduced weight of testis, epididymis, and prostate, a profound alteration of testis histological architecture, together with a significant reduction in epididymal sperm count, motility, and viability [85]. The parallel treatment with ES aqueous extract was associated with a significant improvement in sperm parameters at the lowest dosage of 50 mg/kg, and, most importantly, no significant increase in serum T was observed. Taken together, this evidence suggests a possible direct effect of *ES* extract on testis without the involvement of the HPG axis. Further evaluation of major markers of oxidative stress, such as malondialdehyde and thiol levels quantification in testis and epididymis specimens, showed that BPA exposure was associated with a significant increase in urogenital ROS production. On the other hand, treatment with low doses of *ES* aqueous extract had the highest ROS scavenging effects in these tissues [85].

The possible role of ES as a source of dietary antioxidants is strengthened by other studies using in vivo models of oxidative stress. Nowfel and Al-Okaily, using male rats exposed to oral hydrogen peroxide for 60 days, reported a significant reduction, compared to unexposed controls, in sperm concentration, normal morphology, and viability together with reduced serum catalase activity and no reduction in testosterone levels [88]. The parallel treatment with ES leaves’ ethanolic extract, at a dosage of 300 mg/kg, was associated with a significant recovery of sperm parameters together with the improvement of serum catalase levels [88].

It is important to note that most of the studies recognized higher doses of ES as detrimental to testis function [77,78,80,85]. As suggested by several authors, basal levels of ROS are required for the physiological regulation of sperm function [89]. On these bases, it might be speculated that excessive exposure to antioxidants can exert massive ROS scavenging, resulting in paradoxical redox unbalance and impairment of sperm cell processes.

## 6. Effects of *Eruca sativa* on Male Fertility: Data from Human Models

There are currently very few data about the effect of ES on human models, whether cellular or in vivo. In regard of the antioxidant properties of ES components, Ciccone et al. recently tested the possible effect of erucin, an H_2_S-donor isothiocyanate compound found in ES, on human umbilical vein endothelial cells (HUVECs) challenged with the pro-inflammatory stimulus of lipopolysaccharide (LPS). Interestingly, pre-treatment with 3 mM erucin was able to significantly reduce HUVEC markers of inflammation, such as cell membrane permeability, VE-Cadherin expression (involved in leukocyte adhesion and migration), and cell-mediated ROS production [90]. Interestingly, circulating blood white cells appeared to be a possible target of erucin action, since CD11b-integrin levels in Ly6G+ neutrophils stimulated with LPS were markedly reduced upon pre-treatment with the isothiocyanate compound.

More related to the reproductive field is the study by Grami et al. released in 2018, in which the possible direct effect of ES leaves’ aqueous extract was tested on human spermatozoa from healthy donors exposed to BPA [91]. Interestingly, 10 μM of BPA exposure for 4 hours was able to significantly impair sperm progressive motility compared to unexposed controls. A one-hour pre-treatment with the lowest concentration tested of ES extract (15.6 μg/mL) prevented BPA disruption. Further analysis showed a possible direct effect of ES extract on sperm mitochondria, associated with the recovery of the membrane potential of the organelle, evaluated by a fluorochrome probe, with no major involvement of cell membrane potential [91]. Once again, treatment with higher concentrations of ES extract was associated with the impairment of cell motility, supporting the detrimental role of massive ROS scavenging on sperm function.

## 7. Conclusions

*Eruca Sativa*, whether considering leaves or seeds, has recently drawn attention because of its recognized bioactivity. It is considered a valuable plant in both traditional and modern drug development for its possible medicinal properties. All civilizations have used plants as sources of food due to their essential nutritional value and physiological effects, as well as for their use in pharmaceutical applications. The suggested therapeutic effectiveness of *ES* encompasses a wide range of antioxidant properties believed to be associated with reducing the risk of cardiovascular and cognitive diseases. While efforts are underway to unravel the mechanism behind its actions, a unifying theory is still under investigation.

In traditional oriental medicine, *ES* has been used to enhance male sexual function and restore testicular functions. While some studies in animal models sustain the potential effects of rocket on spermatogenesis and male infertility, available data overall remain scarce. Moreover, human studies on this topic are still lacking, and the beneficial effects of rocket in improving male fertility potential remain to be addressed. These disparities may be attributed to three primary factors: (i) the type of preparations used, (ii) the method of administration, and (iii) the dosage employed. Additionally, the concentration of bioactive components in rocket preparations can vary significantly. Moreover, a strict dose-dependency has been observed, with higher doses potentially leading to adverse effects. Further research is needed to precisely determine the appropriate dosage of *ES* that could serve as a potential tool for nutritional supplementation in the complementary treatment of reproductive disorders.
